# The JAK2 inhibitor TG101209 exhibits anti-tumor and chemotherapeutic sensitizing effects on Burkitt lymphoma cells by inhibiting the JAK2/STAT3/c-MYB signaling axis

**DOI:** 10.1038/s41420-021-00655-1

**Published:** 2021-09-29

**Authors:** Yang Zhang, Ji Li, Haiying Zhong, Xiang Xiao, Zhihua Wang, Zhao Cheng, Cunhong Hu, Guangsen Zhang, Sufang Liu

**Affiliations:** 1 Department of Hematology, The Second Xiangya Hospital, Central South University, Changsha, Hunan 410011 China; 2 Institute of Molecular Hematology, Central South University, Changsha, Hunan 410011 China; 3 Department of Oncology, The Second Xiangya Hospital, Central South University, Changsha, Hunan 410011 China

**Keywords:** Cancer therapy, Cell signalling

## Abstract

Constitutive activation of JAK2/STAT3 is a major oncogenic signaling event involved in the development of Burkitt lymphoma (BL). In the present study, we investigated the antilymphoma activity of TG101209, a specific JAK2 inhibitor, on EBV-positive and EBV-negative Burkitt lymphoma cell lines and primary BL cells. The results showed that TG101209 had a significant antilymphoma effect by inhibiting BL cell growth and inducing apoptosis along with cell differentiation toward mature B cells in vitro. We also found that TG101209 displayed significant synergistic action and a sensitizing effect on the anti-Burkitt lymphoma activity of doxorubicin. In vivo experiments indicated that TG101209 could suppress tumor growth and prolong the overall survival of BL cell-bearing mice. The mechanistic study indicated that TG101209, by suppressing the JAK2/STAT3/c-MYB signaling axis and crosstalk between the downstream signaling pathways, plays an antilymphoma role. These data suggested that TG101209 may be a promising agent or alternative choice for the treatment of BL.

## Background

Burkitt lymphoma (BL) is an aggressive B-cell lymphoma that occurs in children and adults and is largely curable with the use of intensive and toxic chemotherapy. Current treatments are less effective and have more severe side effects in adults and patients with immunodeficiency than in children [1, 2]. BL is often involved in the maxillofacial and abdominal organs, central nervous system and other extranodal organs or in the form of Burkitt leukemia variant, suggesting a higher tumor burden, a fast-growing manner and predisposition to the risk of chemotherapy-related tumor lysis syndrome [3]. BL is also characterized by a translocation involving the myc oncogene. Principles of therapy include high doses of alkylating agents, frequent administration of chemotherapy, and attention to central nervous system (CNS) prophylaxis with high doses of systemic chemotherapy, intrathecal therapy, or both. To date, although the application of short-term intensive chemotherapy combined with the anti-CD20 antibody rituximab has greatly improved the rate of complete remission and overall survival of BL [4], the toxic response or age limitation of chemotherapy is still the main barrier for BL therapy. Therefore, it is urgent and important to find new treatment methods to improve the treatment of Burkitt lymphoma.

The Janus kinase (JAK)/signal transducer and activator of transcription (STAT) pathway is a central signaling pathway by cytokine receptors and is critical in blood lineage development and the immune response. Constitutive activation of STAT pathways may transmit antiapoptotic, proliferative and differentiation signals and contribute to tumor development, invasion and metastasis [5]. Several blood malignancies, including adult T-cell lymphoblastic leukemias [6, 7], B-precursor acute lymphoblastic leukemia [8], and Hodgkin lymphoma [9], have been associated with constitutive activation of STATs. Human myeloproliferative neoplasms (MPNs) were discovered to be associated with a unique acquired somatic mutation in JAK2 (JAK2 V617F [10]) that constitutively activates JAK2. Therefore, developing JAK2 tyrosine kinase inhibitors has become an attractive therapeutic goal for MPNs. Ruxolitinib, a selective Jak2 inhibitor, has been used for the clinical treatment of primary myelofibrosis with the JAK2 V617F mutation [11]. In addition, inhibition of JAK2 kinase may have a therapeutic role in other hematologic malignancies, such as lymphoma and ALL. Because ruxolitinib lacks strict specificity for JAK2 and has drug side effects causing anemia and thrombocytopenia [11], exploring new JAK2 inhibitors for the treatment of hematologic malignancies is urgent and important.

TG101209, a small-molecule JAK2-selective inhibitor, has better JAK2 targeting to primary myelofibrosis than ruxolitinib [12, 13]. TG101209 has obvious cytotoxic effects by arresting cell cycle progression and inducing apoptosis in multiple myeloma cell lines [14]. Additionally, TG101209 can enhance radiotherapy sensitivity in lung cancer models [15], suggesting that the drug may be a good candidate for auxiliary cancer therapy.

Here, we show for the first time the antilymphoma activity of TG101209 on Burkitt lymphoma cell lines and primary BL cells in vivo and in vitro. We demonstrated that TG101209 has significant anti-BL activity by inhibiting cell growth, promoting apoptosis and inducing partial differentiation and exhibited increased chemotherapeutic sensitivity. The mechanism is associated with the inhibition of the JAK2/STAT3/c-MYB signaling pathway. Our results suggest that TG101209 may be an alternative choice for the treatment of BL.

## Results

### TG101209 inhibits the growth of BL cells, induces G2/M cell cycle arrest and has a chemotherapeutic-sensitizing effect

**Fig. 1:**
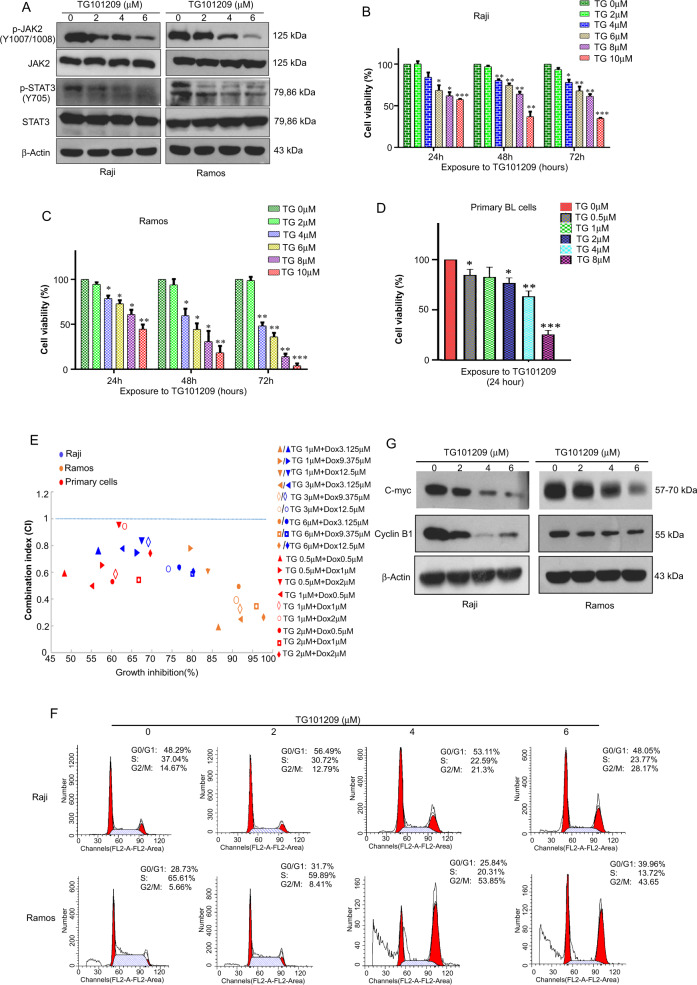
TG101209 inhibits the proliferation of BL cells and induces G2/M cell cycle arrest. **A** Western blot analysis was performed to detect the expression of JAK2, p-JAK2, STAT3 and p-STAT3 in BL cells treated with the indicated concentrations of TG101209 for 0.5 h to confirm the effect of TG101209 on the JAK/STAT3 signaling pathway. Raji (**B**), Ramos (**C**) and primary BL cells (**D**) were treated with TG101209 at different concentrations for indicated time, and cell viability was determined by MTT assay. **E** Combination index (CI) value of TG101209 and doxorubicin was calculated using the method as described in Materials and methods. Drug synergy, addition, and antagonism are defined by CI values less than 1.0, equal to 1.0, or greater than 1.0, respectively. **F** Flow cytometric analysis of the cell cycle in Raji and Ramos cells exposed to TG101209 for 48 h. **G** Western blot analysis was performed to detect the expression of c-Myc and cyclin B1 in BL cells treated with the indicated concentrations of TG101209 for 48 h. The data represent the mean ± SEM of three different experiments. *p < 0.05, **p < 0.01 and ***p < 0.001 compared with the negative control (Student’s *t*-test).

To confirm the inhibitory activity of TG101209 on the constitutive activation of JAK2/STAT3 signaling in BL cells, we detected the phosphorylation status of JAK2/STAT3 in TG101209-treated cells. While phosphorylated Y705 (pY705) is generally believed to be essential for STAT3’s transcriptional activity; we have detected the pY705 of STAT3 to reflect the activity of STAT3.The results showed that TG101209 (6 μM) might further inhibit JAK2 and STAT3 phosphorylation at the half-hour time point in Raji cells or Ramos cells. We also observed that TG101209 could significantly inhibit the phosphorylation of JAK2/STAT3 proteins in a dose-dependent manner (TG101209:0, 1, 2, 4, 6 μM) (Fig. 1A).

The antilymphoma activity of TG101209 was analyzed using MTT and cell cycle distribution assays. The results showed that TG101209 could significantly inhibit the growth of Raji, Ramos and primary BL cells in a dose- or time-dependent manner, with IC50 values of 8.18 μmol/L (Raji), 7.23 μmol/L (Ramos) and 4.57 μmol/L (primary BL cells), respectively (Fig. 1B–D). Our results also showed that when TG101209 was combined with doxorubicin, significant synergistic effects for growth inhibition on BL cells were observed, with a low CI value and a higher proliferation inhibition rate (Fig. 1E, Supplementary Fig. 1 and Supplementary Tables 1–3).

The cell cycle progression assay showed that with increasing TG101209 concentration, the S phase cell ratio of Raji and Ramos cells decreased significantly, and the G2/M phase cell ratio increased. At a concentration of 6 μmol/L, TG101209 blocked most cells in G2/M phase, indicating that TG101209 inhibits cell proliferation and division involved in G2/M cell cycle arrest (Fig. 1F). The regulatory mechanisms of TG101209 in BL cell cycle arrest were related to downregulation of c-myc and cyclinB1 protein expression (Fig. 1G).

### TG101209 induces BL cell apoptosis by a mitochondrial-mediated caspase-dependent pathway

**Fig. 2:**
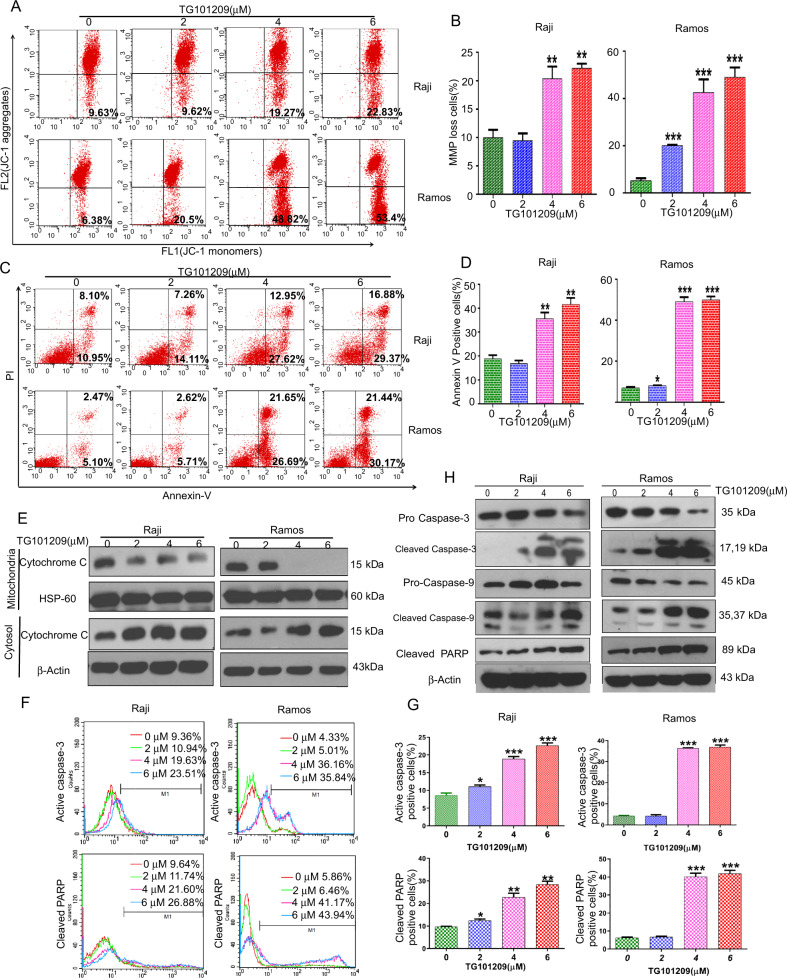
TG101209 induces BL cell apoptosis by a mitochondrial-mediated caspase-dependent pathway. The BL cells were treated with the indicated concentrations of TG101209 for 48 h, then the cells were determined by flow cytometry analysis. **A** The mitochondrial membrane potential (ΔΨm), which was monitored by JC-1 using flow cytometric detection of green fluorescent aggregates in the FL1 channel. **B** The graph shows the percentages of JC-1 monomer (MMP loss) cells. **C** Apoptotic cells treated with TG101209 were determined by flow cytometry analysis of annexin-V FITC and PI. **D** The graph shows the percentages of annexin-V-positive (apoptosis) cells. **E** Mitochondrial/cytosolic cytochrome c expression was assayed by western blotting in TG101209-treated BL cells. HSP60 is a mitochondrial protein marker, and β-actin is a cytosolic fraction marker. **F** The effects of TG101209 on the expression of activated caspase-3 and cleaved PARP were determined using flow cytometry analysis. **G** The graph shows the percentages of activated caspase-3- and cleaved PARP-positive (apoptotic) cells. **H** Effects of TG101209 on pro-caspase-9, pro-caspase-3, cleaved caspase-3, cleaved PARP and cleaved caspase-9 protein expression (western blot). β-Actin was used as a loading control. The data represent the mean ± SEM of three different experiments. **p < 0.01 and ***p < 0.001 compared with the negative control (Student’s *t*-test).

For the detection of early apoptosis, we used cell membrane potential (MMP) measurement and annexin V-FITC/PI assays. The results showed that when Raji and Ramos cells were treated with TG101209 for 48 h, BL cells exhibited significantly reduced membrane potential (Fig. 2A and B), suggesting early apoptosis. The phenomenon of TG101209-induced apoptosis was also confirmed by the annexin V-FITC/PI assay (Fig. 2C and D).

To determine the mechanisms of TG101209-induced BL cell apoptosis, we investigated the distribution of cytochrome c in mitochondria and cytoplasm under TG101209 driving. Our results showed that TG101209 might decrease cytochrome c in mitochondria and increase cytochrome c in the cytoplasm of BL cells in a dose-dependent manner, indicating that cytochrome c was released from mitochondria into the cytoplasm (Fig. 2E). Further study demonstrated that TG101209-induced BL cell apoptosis was accompanied by cleaved caspase-3, caspase-9 and PARP activation (Fig. 2F–H), indicating that TG101209-induced apoptosis of BL cells was dependent on the mitochondrial-mediated caspase pathway.

### TG101209 promotes differentiation potential by downregulating c-MYB expression in Burkitt lymphoma cells

**Fig. 3:**
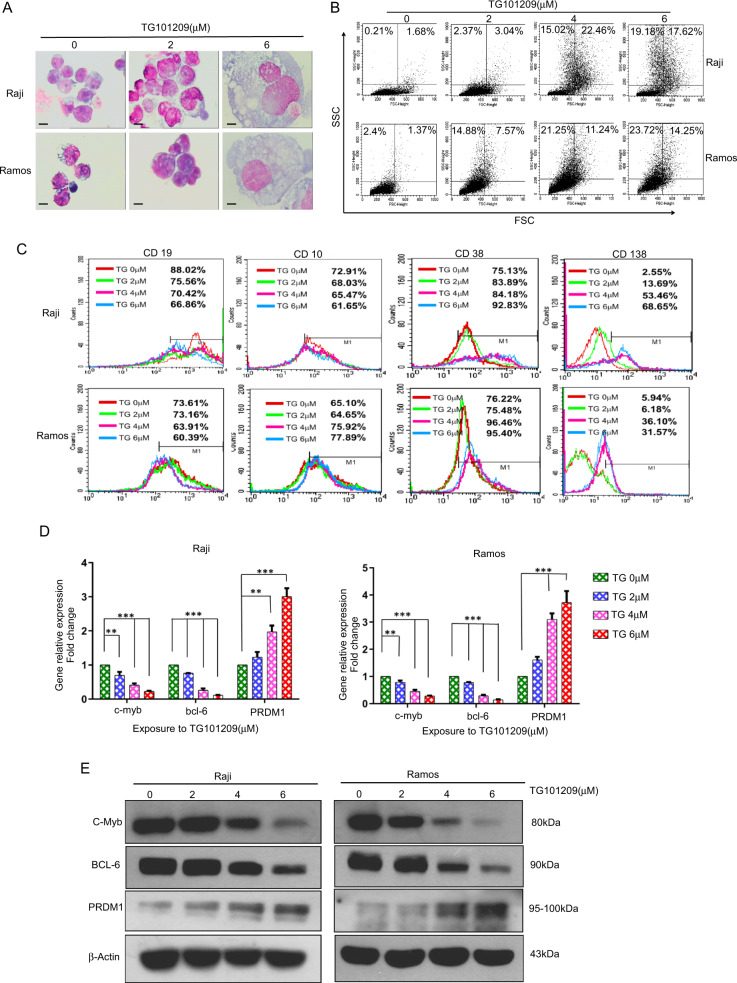
TG101209 promotes differentiation potential by downregulating c-myb expression in Burkitt lymphoma cells. **A** Morphological characteristics of BL cells treated with TG101209 for 96 h were assessed by Wright-Giemsa staining and observed under a Nikon inverted light microscope (Eclipse TE300; Nikon Corporation, Tokyo, Japan) at 1000× magnification. Scale bars, 20 μm. **B** BL cells were treated with the indicated concentrations of TG101209 for 96 h. Flow cytometry was used to detect FSC and SSC. FSC reflects the size of cells, and SSC reflects the number of organelles in cells. **C** The effect of TG101209 on the cluster of differentiation (CD) surface markers of lymphoma cells. Raji and Ramos cells were treated with different concentrations of TG101209 for 96 h. The cells were collected and incubated with specific antibodies against CD19, CD10, CD38 and CD138. Then, the cells were analyzed by flow cytometer. **D** TG101209 regulates the expression of genes involved in cell differentiation. BL cells were treated with the indicated concentrations of TG101209 for 48 h. The relative expression levels of the transcripts for differentiation-related genes were estimated by RT-qPCR. Data were normalized to the amount of β-actin mRNA and are represented as 2ΔΔCT. The data represent the mean ± SEM of three different experiments, and the p values are shown (*p < 0.05, **p < 0.01, ***p < 0.001) (Student’s *t*-test). **E** BL cells were treated with the indicated concentrations of TG101209 for 48 h. Cells were disrupted, and immunoblot analysis was performed to determine the expression of the cell differentiation-related proteins c-myb, bcl-6 and PRDM1. β-Actin was detected as a control to verify equal protein loading. Each experiment repeated at least three times.

To determine whether TG101209 has a pro-differentiation role in BL cells, we demonstrated the pro-differentiation effects of TG101209 on Raji and Ramos cells from three aspects: (1) Cytomorphological observation (Wright Giemsa staining) indicated that after TG101209 treatment, the lymphoma cell volume became larger with enlarged cytoplasmic volume, and the ratio of nucleus to cytoplasm decreased gradually, as well as cytoplasmic staining became grayish - blue with more vacuoles resembling plasma cells (Fig. 3A), suggesting that TG101209 induces cell differentiation into the terminal stage of B lymphocytic cells. (2) The evaluation of the forward scatter (FSC, reflecting the cell size) and side scatter (SSC, reflecting the number of organelles) was based on flow cytometry. The results showed that compared with the untreated cells, TG101209 significantly increased the cell volume and the number of intracellular organelles in a dose-dependent manner (Fig. 3B), which is consistent with the results of Wright Giemsa staining. (3) The assay of lymphoid differential antigen was based on flow cytometry. For lymphoid differential specific markers, we chose B lymphocytic maturation markers, including CD19, CD10, CD38 and CD138, preplasmablast markers, such as CD19^+^, CD10^-^, CD38^++^, and CD138^+/−^, or plasma cells markers, such as CD19^−^, CD10^−^, CD38^+^, and CD138^+^ [16]. The results showed that in Raji cells, the expression of CD19 and CD10 was downregulated in TG101209-treated cells, while the expression of CD38 and CD138 was upregulated in a dose-dependent manner. In Ramos cells, the expression of CD19, CD38 and CD138 was consistent with that in Raji cells, suggesting that TG101209 could induce BL cell differentiation toward mature lymphocytes or plasmocytes (Fig. 3C).

We further probed the mechanism of TG101209-induced differentiation of BL cells. Differentiation-related genes and proteins, including c-Myb, Bcl-6 and PRDM1 (positive regulatory domain zinc finger protein 1), were detected. The results indicated that PRDM1 mRNA levels were significantly increased in TG101209-treated Raji and Ramos cells, while the expression of Bcl-6 and c-Myb mRNA was markedly decreased (RT-qPCR) (Fig. 3D). The Western blot results were consistent with the RT-qPCR results (Fig. 3E).

### The antilymphoma effect of TG101209 is involved in the JAK2/STAT3/c-Myb-mediated signaling pathway

**Fig. 4:**
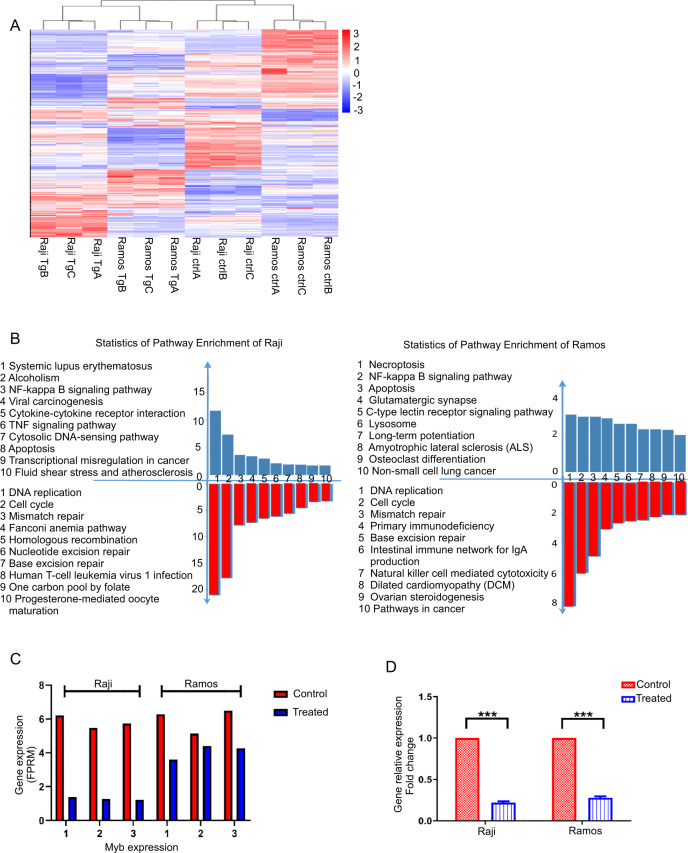
The antilymphoma effect of TG101209 is involved in the JAK2/STAT3/c-Myb-mediated signaling pathway. **A** Hierarchical clustering heatmap of differentially expressed genes between control cells and TG101209-treated cells (*n* = 3). **B** KEGG enrichment analysis of the genes of Raji and Ramos cells between the control group and TG101209-treatment group. Functional enrichment analysis was conducted with the Kyoto Encyclopedia of Genes and Genomes (KEGG) database. **C** Expression levels of c-myb in each sample for RNA sequencing. **D** c-myb gene expression was significantly downregulated in TG101209-treated cells compared with control cells, as revealed by RT-qPCR. The data represent the mean ± SEM of three different experiments. ***p < 0.001 (Student’s *t*-test). FPKM = Fragments per gene per kilobase exon per million mapped reads.

To understand the mechanisms of the antilymphoma activity of TG101209, we performed RNA-seq analysis on TG101209-treated (6 μM) BL cells. Hierarchal clustering analysis indicated that the gene expression patterns in TG101209-treated cells were different from those in untreated cells (Fig. 4A). These up- or downregulated genes were mainly involved in the cell cycle, DNA replication, mismatch repair, natural killer cell-mediated cytotoxicity, and NF-kappa B, TNF and p53 signaling pathways (Fig. 4B). Notably, both TG101209-treated Raji and Ramos cells exhibited commonly involved signaling pathways, including the cell cycle, DNA replication and mismatch repair; however, in Raji cells, the viral carcinogenesis signaling pathway was involved, which was consistent with the biological nature of EB virus-positive Raji cells. Gene expression profiling results also showed that c-Myb was significantly downregulated in TG101209-treated cells (Fig. 4C), which corresponded to the qRT-PCR results (Fig. 4D).

### Knockdown of C-Myb inhibits cell proliferation, arrests cell cycle, promotes apoptosis and cell sensitization to Doxorubicine of BL cells

**Fig. 5:**
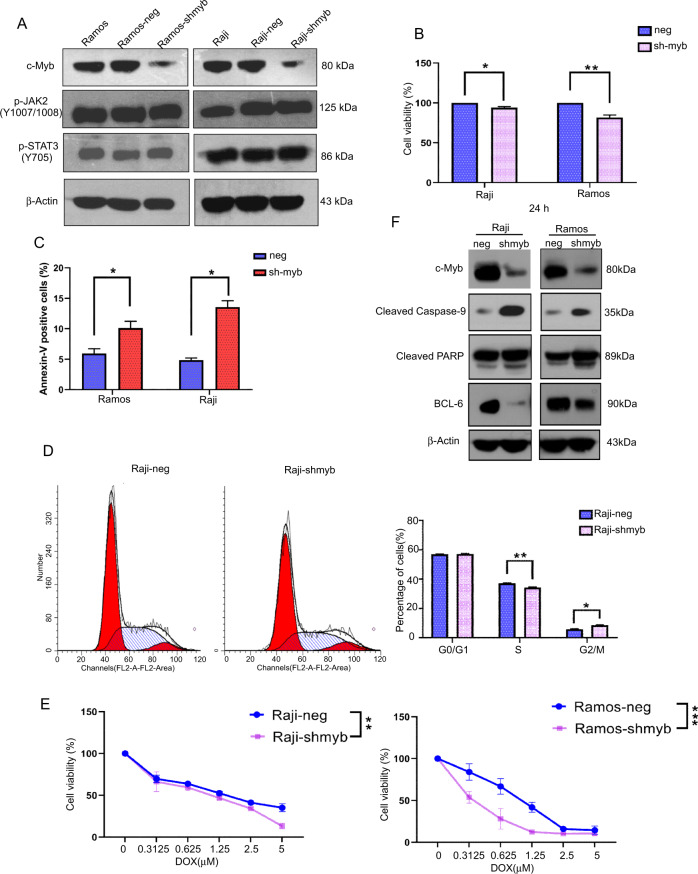
Knockdown of C-Myb inhibits cell proliferation, arrests cell cycle, promotes apoptosis and cell sensitization to Doxorubicine of BL cells. **A** Western blot analysis was performed to detect the expression levels of c-Myb, p-JAK2 and p-STAT3 in Raji and Ramos cells transfected with myb-knockdown plasmid or vector control. **B** Raji and Ramos cells transfected with myb-knockdown plasmid or vector control were seeded in 96-well plate for 24 h, then OD was measured by the method of MTT. **C** Apoptotic cells were determined by flow cytometry analysis of annexin-V APC and 7-AAD, and the graph shows the percentages of annexin-V-positive (apoptotic) Raji and Ramos cells transfected with myb-knockdown plasmid or vector control. **D** Flow cytometric analysis of the cell cycle in Raji and Ramos cells transfected with myb-knockdown plasmid or vector control. **E** Raji and Ramos cells transfected with myb-knockdown plasmid or vector control were seeded in 96-well plate were treated with Doxorubicin at different concentrations for 24 h, and cell viability was determined by MTT assay. **F** Western blot analysis was performed to detect the expression levels of cleaved caspase-9, cleaved PARP and Bcl-6 in Raji and Ramos cells transfected with myb-knockdown plasmid or vector control. Data are presented as the mean ± SD, and the p values are shown (*p < 0.05, **p < 0.01, ***p < 0.001).

The above results showed that TG101209 could suppress c-Myb expression. Thus, we then assessed whether knockdown of c-Myb had the same antilymphoma effect as TG101209. We generated c-Myb knockdown Raji and Ramos cell models. Compared with mimic control cells, the expression of c-Myb protein in sh-Myb cells was significantly decreased, but the phosphorylation levels of JAK2 and STAT3 were not affected, suggesting that c-Myb was downstream of the JAK2/STAT3 signaling pathway (Fig. 5A). In order to examine the role of c-Myb on BL cells proliferation, we examined the effect of sh-Myb on BL cells growth and spontaneous apoptosis. As shown in Fig. 5B, sh-Myb significantly decreases cell proliferation in Raji and Ramos cells at 24 h compared to control group, respectively. The results (annexin V-APC/7-AAD) showed that c-Myb knockdown itself led to a marked increase in cell apoptosis (Fig. 5C). To reveal mechanism involved in proliferation inhibition, we analyzed cell cycle by using flow cytometry. As shown in Fig. 5D, knockdown of c-Myb resulted in accumulation in the G2/M phase and reduction of S phase cell. Furthermore, we tested if knock down c-Myb could sensitize BL cells to chemotherapeutic agents. Raji and Ramos cells were exposed to doxorubicin for 24 h after transfected with sh-myb. Cells’ viability was evaluated using a MTT assay. As shown in Fig. 5E, cells transfected with sh-Myb were more sensitive to doxorubicin than control. Western blotting showed that knockdown of c-Myb dramatically induced the cleavage of caspase-9 and PARP and inhibited the expression of bcl-6 in Raji and Ramos cells transfected with sh-Myb (Fig. 5F), indicating that c-Myb is required for antiapoptotic potency in Raji and Ramos cells and that c-Myb might be a necessary endogenous target involved in the antilymphoma activity of TG101209.

### TG101209 inhibits proliferation of Ramos cells in vivo

**Fig. 6:**
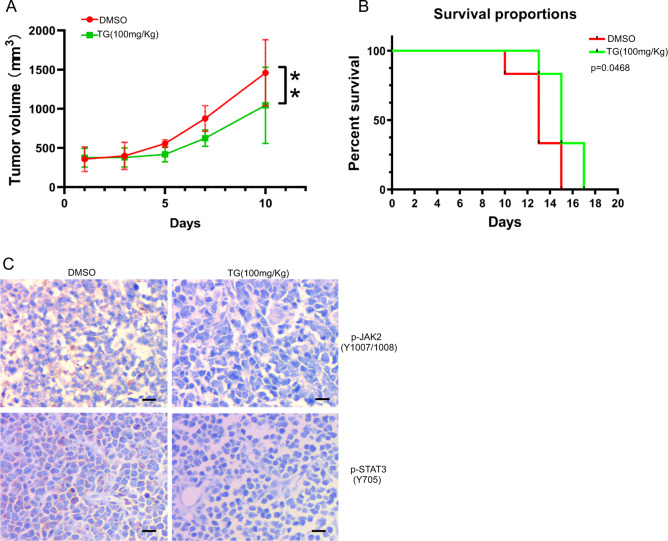
TG101209 inhibits tumor growth in vivo. The nude mice bearing Ramos-derived tumor xenografts were orally gavaged with TG101209 at doses of 100 mg/kg BID for 7 consecutive days (*n* = 6 per group), whereas the control group received DMSO only. **A** Tumor volume curves showed that TG101209 significantly suppressed the growth of tumor xenografts. **B** The Kaplan–Meier method was used for survival analysis, and the log rank test was used to compare the survival rate of the two groups. *p < 0.05. **C** Expression levels of p-JAK2 and p-STAT3 in the tumors from mice treated with TG101209 or vehicle were detected by IHC staining. Original magnification ×400. Scale bars, 100 μm.

Nude mice bearing Ramos-derived tumor xenografts were orally administered TG101209 (100 mg/kg), and the antilymphoma effect of TG101209 was monitored. The results showed that TG101209 significantly inhibited tumor growth in xenograft models (Fig. 6A). The difference in overall survival between the TG101209-treated mice and the untreated mice was statistically significant (*P* < 0.05; log-rank analysis) (Fig. 6B). IHC images showed that TG101209 inhibited p-JAK2/p-Stat3 expression, which was consistent with the in vitro results (Fig. 6C).

## Discussion

Abnormal activation of the JAK2/STATS signaling pathway is closely related to the occurrence and development of lymphoma. Some data have confirmed that the expression of JAK2 is abnormally increased in approximately 30% of Hodgkin’s lymphoma (HL) and 30–50% of primary mediastinal large B-cell lymphoma (PMBCL), suggesting that the abnormal activation of JAK2 is associated with the development of lymphoma [17, 18]. With the downstream targeting of JAK2, the persistent activation of STAT3 in primary exudative lymphoma (PEL) and large granular lymphocytic leukemia (LGL) has been revealed, where approximately 40% of LGL subtypes have increased STAT3 activity and are used as a diagnostic marker of LGL [19, 20], implying that, to a certain extent, targeting the JAK2/STAT3 signaling pathway for lymphoma therapy is reasonable. More recently, ruxolitinib has broadened its therapeutic indications, including preventing and controlling acute and chronic steroid-refractory graft-versus-host disease (srGVHD) [21], successfully treating the deficiency of ubiquitin-specific peptidase 18 (USP18)-associated severe type I interferonopathy accompanied by systemic inflammation and respiratory failure [22], and partially reverses functional natural killer cell deficiency in patients with STAT1 gain-of-function mutations [23].

In the present study, we chose TG101209, a more specific or selective JAK2 kinase inhibitor (at approximately 30-fold greater selectivity for JAK2 than JAK3) [12, 13], as an exploration of anti-Burkitt lymphoma activity. Our results showed that TG101209 can significantly inhibit the proliferation of EBV-positive (Raji) (IC50: 8.18 μmol/L) and EBV-negative (Ramos) (IC50: 7.23 μmol/L) BL cells, as well as primary cells from the bone marrow of one patient with Burkitt lymphoma, in a dose-dependent manner. Interestingly, we also observed a significantly synergistic effect of antilymphoma activity when TG101209 was combined with doxorubicin (a main drug for anti-acute lymphoblastic leukemia or lymphoma), suggesting that TG101209 may be an alternate choice for Burkitt lymphoma therapy. It has been reported that inhibition of the JAK/STAT pathway using of JAK1/2 inhibitors have demonstrated promising results and limited side effects [24, 25]. Our in vivo experimental results displayed a strong antilymphoma effect on Ramos cell xenografts and significantly inhibited tumor growth and prolonged overall survival of Ramos cell-bearing mice, without apparent side effects. The mechanisms of the antiproliferative role of TG101209 were related to cell cycle arrest in G2/M phase. Overexpression of c-Myc is involved in the tumorigenesis of B-lineage acute lymphoblastic leukemia (BALL). A c-Myc knockdown model (Raji cells) showed downregulated cyclin-dependent kinases (CDK) 1 and cyclin B1 expression, which were responsible for cell cycle progression in G2/M phase [26]. These results were consistent with our observation that TG101209 might markedly suppress the expression of c-Myc and cyclin B1 in Raji and Ramos cells, resulting in G2/M arrest. Therefore, blocking the c-Myc/cyclin B1 signaling pathway may be one of the important mechanisms for the antiproliferative activity of TG101209.

Induction of apoptosis is a common strategy and mechanism in anticancer therapy. Cillessen SA et al reported that patients with primary nodal diffuse large B-cell lymphomas (DLBCLs) are correlated with the expression of inhibitors of the intrinsic apoptosis pathway, including X-linked inhibitor of apoptosis protein (XIAP). XIAP suppresses apoptosis by inhibiting active caspase-3, caspase-7, and caspase-9. They also showed that the small-molecule XIAP antagonist 1396-12 might induce cell death in cultured primary DLBCL cells by relieving caspase-3 inhibition and constitutive caspase-9 activation [27]. Pardanani A, et al showed TG101209 (600 nM) could induce significant JAK2V617F-expressing HEL and Ba/F3 cells apoptosis with time-dependent manner, suggesting the anti-leukemic cells activity of TG101209 is associated with cell cycle arrest and induction of apoptosis [12]. Ramakrishnan, V et al studied the effects of TG101209 on CD45 positive myeloma cell lines and primary plasma cells from myeloma patient, and confirmed that the induction of cytotoxicity of TG101209 accompanied by inhibition of cell cycle progression and induction of apoptosis, in which the mechanism of action of TG101209 involved in down regulation of p-JAK2, p-STAT3 and Bcl-xl levels [14]. In the present studies, we first showed in BL cells, that TG101209 inhibited proliferation of BL cells through cell cycle effects and induction of apoptosis. The mechanisms related to the down regulation of c-myc and cyclinB1 levels in cells cycle progression and involved in an intrinsic (mitochondrial-dependent) apoptotic pathway [28], in which the features of intrinsic apoptotic pathway, including decreased mitochondrial membrane potential and increased cytochrome c release, and the molecular markers of apoptosis activation, such as caspase-9, caspase-3 and RAPR cleavage.

Cell differentiation disorder is an important biological feature of tumor cells. At present, the JAK2 inhibitors are gradually becoming a research hot spot in the field of tumor treatment, especially in hematological malignancies. These studies were performed to focus on the effects of the suppression tumor growth, promoting apoptosis of tumor cells, inhibiting angiogenesis and metastasis. However, very few studies have been conducted to evaluate the efficacy of JAK2 inhibitors in anti-Burkitt lymphoma and inducing the lymphoma cells differentiation. Considering that TG101209 is a small molecular kinase inhibitor, its antilymphoma activity is not only dependent on the cytotoxic effect of the drug but may also be involved in promoting differentiation. We wanted to determine whether TG101209 has pro-differentiation potential in BL cells. Our results demonstrated that TG101209 could induce BL cell differentiation toward mature lymphoid or plasmacytoid cells with the morphological features of differentiation and enhanced CD138 and CD38 expression and weaker CD19 and CD10 expression (Fig. 3). To probe the mechanism by which TG101209 induces BL cell differentiation, we investigated the changes in differentiation-related genes, including Bcl-6, c-Myb and PRDM1. The results showed that TG101209 significantly inhibited the expression of Bcl-6 and c-Myb mRNA or protein and enhanced the expression of PRDM1, suggesting that blocking c-Myb/PRDM1/Bcl-6 signaling contributed to unblocking the arrest of BL cell differentiation. Previous investigation has indicated c-MYB was not down-modulated by 0.6 µM or 1.2 µM TG101209 in the JAK2V617F cell lines SET2 and UKE1 [29]. The discrepancy between the previous findings with the present results may be due to differences in the experimental systems and the concentration of TG101209. Recently, studies have shown that oncogenic c-Myb can enhance the activity of T-cell acute lymphoblastic leukemia [30]. Dysregulation of the c-Myb pathway provides the basis for adult T-cell leukemia/lymphoma cells [31], and high expression of c-Myb in tumor tissues may be a predictor of poor prognosis for Burkitt lymphoma patients [32], illustrating that c-Myb dysregulation is actively involved in the pathogenesis and prognosis of lymphoid tumors. Several studies have reported that BCL6 and PRDM1 are important regulatory genes in the differentiation of B cells and are closely related to the differentiation of terminal B lymphocytes. As a transcription inhibitor, BCL-6 regulates the differentiation of germinal center B lymphocytes by inhibiting the expression of PRDM1 and preventing germinal center B cells from differentiating into plasma cells [33]. PRDM1, also known as Blimp-1 (B lymphocyte induced maturation protein 1) protein, is an important immune marker of plasma cell differentiation. By inhibiting the expression of BCL6 [34], PRDM1 promotes B lymphocyte differentiation into plasma cells, suggesting that PRDM1 is a switch gene for plasma cell differentiation [33, 35, 36]. Combined with our data, we demonstrated that the pro-differentiation role of TG101209 is related to the c-MYB/PRDM1/Bcl-6 signaling axis.

**Fig. 7:**
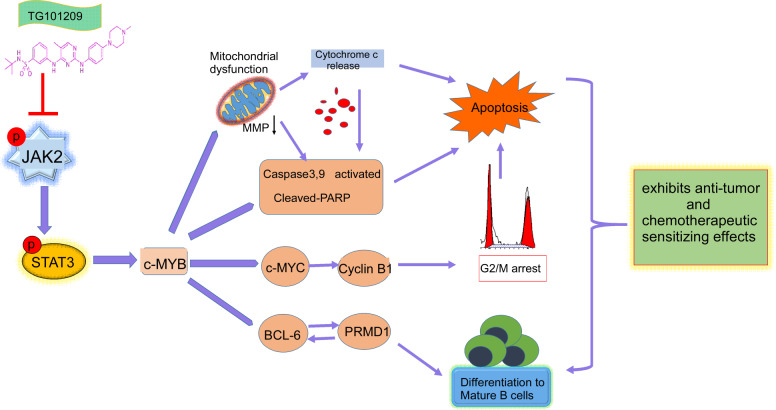
Proposed model summarizing the results. TG101209 exhibits the activity of anti-tumor and chemotherapeutic sensibilizating effect on Burkitt lymphoma cells by inhibiting the JAK2/STAT3/c-MYB signaling axis.

C-Myb is highly expressed or dysregulated in immature hematopoietic cells, hematologic tumors and several solid tumors [30–32, 37–39]. In earlier initial lymphocytes, c-Myb deletion can prevent the transition of initial lymphocytes to pre-B lymphocytes, thus blocking the differentiation and maturation of B lymphocytes. High expression of c-Myb can also lead to the blockade of terminal differentiation and the malignant transformation of B lymphocytes [40] and enhance ovarian cancer cell proliferation, invasion, and cisplatin resistance [41], suggesting that c-Myb plays an important role in regulating the biological behavior and chemotherapeutic resistance of malignant tumors. We also showed higher expression of both c-Myb mRNA and protein in BL cells, and knockdown of c-Myb in Raji and Ramos cells might induce the cleavage activation of apoptotic molecules (caspase-9, PARP) and inhibit Bcl-6 protein levels, illustrating that c-Myb plays an important role in apoptosis resistance in BL cells. Altogether, the main mechanisms of anti-BL activity for TG101209 are associated with the interruption of the JAK2/STAT3/c-MYB signaling pathway. When the chief axis of JAK2/STAT3 signaling is suppressed, c-MYB, as a downstream core signal, regulates the differentiation, growth and apoptosis of BL cells by crosstalk with the Bcl-6/PRDM1, c-Myc/cyclin B1 and apoptotic signaling pathways (Fig. 7).

In brief, we showed for the first time that TG101209, a specific JAK2 inhibitor, could inhibit Burkitt lymphoma growth, induce cycle arrest, differentiation and apoptosis of BL cells, and prolong the survival of Ramos cell-bearing mice by inhibiting the JAK2/STAT3/c-MYB signaling axis. Additionally, TG101209 displayed an effect of chemotherapy sensitization on BL cells. Therefore, TG101209 may be a potential therapeutic agent and alternative choice for the treatment of Burkitt lymphoma.

## Materials and methods

### Patient sample, cell lines and reagents

Bone marrow samples from a patient with Burkitt leukemia variant [42] were obtained at our department, and bone marrow mononuclear cells (BMMNCs) were isolated. The EBV-positive Burkitt lymphoma cell line (Raji, JAK2 wild-type) and EBV-negative BL cell line (Ramos, JAK2 wild-type) were kind gifts from Prof. Y. Cao (Institute of Oncology, Central South University, Changsha, China). Both cell lines and primary BMMNCs were cultured in RPMI 1640 medium (Gibco, Grand Island, NY, USA) with 10% fetal calf serum under humidified air containing 5% CO2 at 37 °C. TG101209 (#S2692;) and doxorubicin (#S1208) were purchased from Selleckchem (Houston, USA) and dissolved in DMSO for stock solutions (10 mmol/L and 2 mmol/L, respectively). The study was approved by the Ethical Committee at the Second Xiangya Hospital, Central South University, and the patient gave written informed consent according to the Declaration of Helsinki.

### Cell viability assay by MTT colorimetric method

The 3-(4, 5-dimethylthiazole-2-yl)-2, 5-diphenyltetrazolium bromide (MTT) assay was used to assess the effects of TG101209 on cell viability as described previously [43]. Briefly, cells (5 × 10^4^ per well) were plated in triplicate wells with 200 μL RPMI 1640 medium supplemented with 10% FBS in 96-well microplates and treated with different concentrations of TG101209 for 24, 48 or 72 h. Cell viability was evaluated by MTT assay. To observe the synergistic effect of antilymphoma activity, TG101209 (1, 3, and 6 μM) and doxorubicin (3.125, 9.375, and 12.5 μM) were added to BL cells alone or in combination. IC50 values were determined using the nonlinear regression program CalcuSyn (Cambridge, UK).

### Cell apoptosis measurements using annexin V staining and mitochondrial transmembrane potential

Raji and Ramos cells were seeded in 6-well plates and incubated with various doses of TG101209 (0, 2, 4, 6 μM) for 48 h. The mitochondrial membrane potential (MMP, ΔΨ_m_) was determined using a JC-1 Apoptosis Detection Kit (KeyGEN BioTECH, China) according to the manufacturer’s instructions, and the fluorescence of JC-1-carrying cells was measured by a FACSCalibur flow cytometer with FL1 and FL2 channels. Furthermore, apoptosis was also assessed with an Annexin V-FITC/propidium iodide (PI) apoptosis detection kit (KeyGEN BioTECH, China) by flow cytometry. Apoptotic cells of Raji and Ramos cells transfected with myb-knockdown plasmid or vector control were determined by flow cytometry analysis of annexin-V APC and 7-AAD apoptosis detection kit (KeyGEN BioTECH, China).

### Cell differentiation analysis

Raji and Ramos cells were treated with 6 μM TG101209 for 96 h and then incubated with FITC-labeled anti-CD38 antibody (#555459), PE-labeled anti-CD138 (#552026), APC-labeled anti-CD19 (#555415) and PE-labeled anti-CD10 (#555375) (BD Pharmingen, CA), and the percentage of positive cells was examined by flow cytometry. For morphological analysis, cytospin slides of each sample were stained with Wright-Giemsa staining and observed under a Nikon inverted light microscope (Eclipse TE300; Nikon Corporation, Tokyo, Japan) at 1000 × magnification. Physical parameters (forward scatter [FS] and side scatter [SS]) in Raji and Ramos cell lines were also analyzed by flow cytometry.

### Western blot

Raji and Ramos cells were treated with various doses of TG101209 (0, 2, 4, 6 μM) for 24–48 h. Then the total protein, mitochondrial fractions and cytosolic fractions were prepared, and the protein concentration was determined using BCA Assay Reagent (Pierce, Rockford, IL). For the preparation of mitochondrial or cytosolic fractions, mitochondria were isolated using a mitochondria isolation kit (Thermo Fisher Scientific, Waltham, MA, USA) following a previous report [44]. Equal amounts of protein were separated by 8% or 12% SDS-PAGE and transferred onto PVDF membranes. After blocking the membranes with 5% fat-free milk, protein signals were probed with various primary antibodies and HRP-conjugated secondary antibodies (see Supplementary Information) and visualized with enhanced chemiluminescence detection reagents (Thermo Fisher Scientific, Waltham, MA, USA).

### Quantitative reverse transcription polymerase chain reaction

Raji and Ramos cells were treated with various doses of TG101209 (0, 2, 4, 6 μM) for 48 h. Total RNA was extracted from cells using TRIzol reagent (Takara Bio, Inc., Otsu, Japan) according to the manufacturer’s protocols. Primer sequences for Bcl-6, PRDM1, c-Myb and β-actin messenger RNA (mRNA) are available in the Supplementary Table 4. Complementary DNA was synthesized using 1–5 μg of purified RNA, the RevertAid™ First Strand cDNA Synthesis Kit (Fermentas, EU) and Oligo (dT). Quantitative reverse transcription PCR (qRT-PCR) was performed on a Rotor-Gene 6000 PCR instrument. Relative mRNA abundance was calculated using β-actin as an internal control using the 2ΔΔCT method. The mRNA changes are represented relative to untreated cells.

### Gene expression profiling and data analysis

Raji and Ramos cells were treated with DMSO or 6 μM TG101209 for 48 h, and total RNA was extracted. TG101209-treated samples or DMSO-control samples were analyzed on the BGISEQ-500 platform. Microarray hybridization was performed according to the manufacturer’s protocols. RNA-seq data were generated by Novogene (Novogene Bioinformatics Technology Co., Ltd., Beijing, China). Differentially expressed genes between two samples were identified through fold-change filtering (≥2.0). Pathway analysis was performed with gene mapping to KEGG pathways (*P* < 0.05). Finally, hierarchical clustering was used to distinguish gene expression among samples.

### siRNA-mediated RNA interference

Double-stranded siRNA (RiboBio) to silence endogenous expression in Raji and Ramos cells targeted human c-Myb mRNA (sequence: ccAGATTGTAAATGCTCATTT). c-Myb shRNA lentiviral and lentiviral scrambled negative controls were designed and constructed by GeneChem Corporation (Shanghai, China), and the lentiviral supernatant was synthesized by GeneChem. For lentivirus infection, Raji and Ramos cells were seeded in six-well plates and infected by HiTransG A (Genechem) according to the manufacturer’s protocol. Then, Raji and Ramos cells were selected with puromycin for 2 weeks to remove uninfected cells and obtain stable c-myb knocking down cells. The stable c-myb knocking down cells was validated by Western blotting and/or qPCR.

### Ramos-derived xenograft mouse model

The antilymphoma effect of TG101209 in vivo was assessed in nude immunodeficient mice engrafted with Ramos cells. All mice were maintained and manipulated according to strict guidelines established by the Medical Research Animal Ethics Committee, Central South University, China. Ramos cells (1 × 10^7^/100 μL), suspended in RPMI 1640 medium and inoculated into the right flank of 5- to 6-week-old female mice by subcutaneous injection. The mice were categorized randomly into TG101209-treated group and DMSO-treated group (6 mice per group). TG101209 (100 mg/kg) or DMSO was administered daily by oral gavage until the tumor volume reached 100 mm^3^ [15]. Every two or three days, the tumor diameter was measured, and the volume was calculated (length × width 2 × 0.5). Mice were euthanized when the tumor volume reached 1.5 cm^3^_,_ and the study was stopped when more than half of the mice were sacrificed.

### Immunohistochemical (IHC) staining

IHC staining was performed as described previously [45], and the complete procedure is to be found in the Supplementary Information.

### Statistical analysis

All data were expressed as the mean ± SEM. All statistical parameters were calculated in GraphPad Prism software. Significant differences were determined by Student’s *t*-test or ANOVA in comparisons between two groups. For the evaluation of synergistic action, the combination index (CI) values were calculated using CalcuSyn Biosoft software. The overall survival of mice was evaluated by Kaplan–Meier curves using the log-rank test. *P* < 0.05 was considered statistically significant.

## Supplementary information

Supplementary Figure

Supplementary Figure legend

Supplementary tables

Supplementary Information

## Data Availability

Data supporting the findings of this study are available within the article and its supplementary information files.

## References

[1] Dunleavy K, Pittaluga S, Shovlin M, Steinberg SM, Cole D, Grant C (2013). Low-intensity therapy in adults with Burkitt’s lymphoma. N Engl J Med.

[2] Dunleavy K, Little RF, Wilson WH (2016). Update on Burkitt lymphoma. Hematol/Oncol Clin North Am.

[3] Perkins AS, Friedberg JW Burkitt lymphoma in adults. Hematology American Society of Hematology Education Program 2008;341–8. 10.1182/asheducation-2008.1.341 19074108

[4] Hoelzer D, Walewski J, Dohner H, Viardot A, Hiddemann W, Spiekermann K (2014). Improved outcome of adult Burkitt lymphoma/leukemia with rituximab and chemotherapy: report of a large prospective multicenter trial. Blood.

[5] Bowman T, Garcia R, Turkson J, Jove R (2000). STATs in oncogenesis. Oncogene.

[6] Takemoto S, Mulloy JC, Cereseto A, Migone TS, Patel BK, Matsuoka M (1997). Proliferation of adult T cell leukemia/lymphoma cells is associated with the constitutive activation of JAK/STAT proteins. Proc Natl Acad Sci USA.

[7] Cheng Z, Yi Y, Xie S, Yu H, Peng H, Zhang G (2017). The effect of the JAK2 inhibitor TG101209 against T cell acute lymphoblastic leukemia (T-ALL) is mediated by inhibition of JAK-STAT signaling and activation of the crosstalk between apoptosis and autophagy signaling. Oncotarget.

[8] Tasian SK, Doral MY, Borowitz MJ, Wood BL, Chen IM, Harvey RC (2012). Aberrant STAT5 and PI3K/mTOR pathway signaling occurs in human CRLF2-rearranged B-precursor acute lymphoblastic leukemia. Blood.

[9] Vainchenker W, Constantinescu SN (2013). JAK/STAT signaling in hematological malignancies. Oncogene.

[10] Kralovics R, Passamonti F, Buser AS, Teo SS, Tiedt R, Passweg JR (2005). A gain-of-function mutation of JAK2 in myeloproliferative disorders. N Engl J Med.

[11] Tefferi A (2012). Challenges facing JAK inhibitor therapy for myeloproliferative neoplasms. N Engl J Med.

[12] Pardanani A, Hood J, Lasho T, Levine RL, Martin MB, Noronha G (2007). TG101209, a small molecule JAK2-selective kinase inhibitor potently inhibits myeloproliferative disorder-associated JAK2V617F and MPLW515L/K mutations. Leukemia.

[13] Wang Y, Fiskus W, Chong DG, Buckley KM, Natarajan K, Rao R (2009). Cotreatment with panobinostat and JAK2 inhibitor TG101209 attenuates JAK2V617F levels and signaling and exerts synergistic cytotoxic effects against human myeloproliferative neoplastic cells. Blood.

[14] Ramakrishnan V, Kimlinger T, Haug J, Timm M, Wellik L, Halling T (2010). TG101209, a novel JAK2 inhibitor, has significant in vitro activity in multiple myeloma and displays preferential cytotoxicity for CD45+ myeloma cells. Am J Hematol.

[15] Sun Y, Moretti L, Giacalone NJ, Schleicher S, Speirs CK, Carbone DP (2011). Inhibition of JAK2 signaling by TG101209 enhances radiotherapy in lung cancer models. J Thorac Oncol.

[16] Jourdan M, Caraux A, De Vos J, Fiol G, Larroque M, Cognot C (2009). An in vitro model of differentiation of memory B cells into plasmablasts and plasma cells including detailed phenotypic and molecular characterization. Blood.

[17] Derenzini E, Younes A (2013). Targeting the JAK-STAT pathway in lymphoma: a focus on pacritinib. Expert Opin Investig Drugs.

[18] Green MR, Monti S, Rodig SJ, Juszczynski P, Currie T, O’Donnell E (2010). Integrative analysis reveals selective 9p24.1 amplification, increased PD-1 ligand expression, and further induction via JAK2 in nodular sclerosing Hodgkin lymphoma and primary mediastinal large B-cell lymphoma. Blood.

[19] Aoki Y, Feldman GM, Tosato G (2003). Inhibition of STAT3 signaling induces apoptosis and decreases survivin expression in primary effusion lymphoma. Blood.

[20] Koskela HL, Eldfors S, Ellonen P, van Adrichem AJ, Kuusanmaki H, Andersson EI (2012). Somatic STAT3 mutations in large granular lymphocytic leukemia. N Engl J Med.

[21] Moiseev IS, Morozova EV, Bykova TA, Paina OV, Smirnova AG, Dotsenko AA (2020). Long-term outcomes of ruxolitinib therapy in steroid-refractory graft-versus-host disease in children and adults. Bone Marrow Transplant.

[22] Alsohime F, Martin-Fernandez M, Temsah MH, Alabdulhafid M, Le Voyer T, Alghamdi M (2020). JAK inhibitor therapy in a child with inherited USP18 deficiency. N Engl J Med.

[23] Vargas-Hernandez A, Mace EM, Zimmerman O, Zerbe CS, Freeman AF, Rosenzweig S (2018). Ruxolitinib partially reverses functional natural killer cell deficiency in patients with signal transducer and activator of transcription 1 (STAT1) gain-of-function mutations. J Allergy Clin Immunol.

[24] Jones RS, Minogue AM, Fitzpatrick O, Lynch MA (2015). Inhibition of JAK2 attenuates the increase in inflammatory markers in microglia from APP/PS1 mice. Neurobiol Aging.

[25] Kontzias A, Laurence A, Gadina M, O’Shea JJ (2012). Kinase inhibitors in the treatment of immune-mediated disease. F1000 Med Rep.

[26] Yang Y, Xue K, Li Z, Zheng W, Dong W, Song J (2018). c-Myc regulates the CDK1/cyclin B1 dependentG2/M cell cycle progression by histone H4 acetylation in Raji cells. Int J Mol Med.

[27] Cillessen SA, Reed JC, Welsh K, Pinilla C, Houghten R, Hooijberg E (2008). Small-molecule XIAP antagonist restores caspase-9 mediated apoptosis in XIAP-positive diffuse large B-cell lymphoma cells. Blood.

[28] Martin DN, Baehrecke EH (2004). Caspases function in autophagic programmed cell death in Drosophila. Development.

[29] Amaru Calzada A, Todoerti K, Donadoni L, Pellicioli A, Tuana G, Gatta R (2012). The HDAC inhibitor Givinostat modulates the hematopoietic transcription factors NFE2 and C-MYB in JAK2(V617F) myeloproliferative neoplasm cells. Experimental hematology.

[30] Choi A, Illendula A, Pulikkan JA, Roderick JE, Tesell J, Yu J (2017). RUNX1 is required for oncogenic Myb and Myc enhancer activity in T-cell acute lymphoblastic leukemia. Blood.

[31] Nakano K, Uchimaru K, Utsunomiya A, Yamaguchi K, Watanabe T (2016). Dysregulation of c-Myb pathway by aberrant expression of proto-oncogene MYB provides the basis for malignancy in adult T-cell leukemia/lymphoma cells. Clin Cancer Res.

[32] Ma M, Zhao R, Yang X, Zhao L, Liu L, Zhang C (2018). Low expression of Mda-7/IL-24 and high expression of C-myb in tumour tissues are predictors of poor prognosis for Burkitt lymphoma patients. Hematology.

[33] Martinez MR, Corradin A, Klein U, Alvarez MJ, Toffolo GM, di Camillo B (2012). Quantitative modeling of the terminal differentiation of B cells and mechanisms of lymphomagenesis. Proc Natl Acad Sci USA.

[34] Tunyaplin C, Shaffer AL, Angelin-Duclos CD, Yu X, Staudt LM, Calame KL (2004). Direct repression of prdm1 by Bcl-6 inhibits plasmacytic differentiation. J Immunol.

[35] Diehl SA, Schmidlin H, Nagasawa M, van Haren SD, Kwakkenbos MJ, Yasuda E (2008). STAT3-mediated up-regulation of BLIMP1 Is coordinated with BCL6 downregulation to control human plasma cell differentiation. J Immunol.

[36] Crotty S, Johnston RJ, Schoenberger SP (2010). Effectors and memories: Bcl-6 and Blimp-1 in T and B lymphocyte differentiation. Nat Immunol.

[37] Azim S, Zubair H, Srivastava SK, Bhardwaj A, Zubair A, Ahmad A (2016). Deep sequencing and in silico analyses identify MYB-regulated gene networks and signaling pathways in pancreatic cancer. Sci Rep.

[38] Wang Y, Fang R, Cui M, Zhang W, Bai X, Wang H (2017). The oncoprotein HBXIP upregulates YAP through activation of transcription factor c-Myb to promote growth of liver cancer. Cancer Lett.

[39] Yang H, Zhang H, Ge S, Ning T, Bai M, Li J (2018). Exosome-derived miR-130a activates angiogenesis in gastric cancer by targeting C-MYB in vascular endothelial cells. Mol Ther.

[40] Chen S, Wang Z, Dai X, Pan J, Ge J, Han X (2013). Re-expression of microRNA-150 induces EBV-positive Burkitt lymphoma differentiation by modulating c-Myb in vitro. Cancer Sci.

[41] Tian M, Tian D, Qiao X, Li J, Zhang L (2019). Modulation of Myb-induced NF-kB -STAT3 signaling and resulting cisplatin resistance in ovarian cancer by dietary factors. J Cell Physiol.

[42] Leoncini L, Campo E, Harris NL, Jaffe EL, Pileri SA, Stein H, et al. Burkitt lymphoma In: Swerdlow SH, Campo E, Harris NL, et al. editors. WHO Classification of Tumours of Haematopoietic and Lymphoid Tissues. Revised fourth ed. Lyon: International Agency for Research on Cancer (IARC) 2017:330–4.

[43] Xiao X, Jiang K, Xu Y, Peng H, Wang Z, Liu S (2019). (-)-Epigallocatechin-3-gallate induces cell apoptosis in chronic myeloid leukaemia by regulating Bcr/Ablmediated p38-MAPK/JNK and JAK2/STAT3/AKT signalling pathways. Clin Exp Pharmacol Physiol.

[44] Wang Z, Zhang Y, Zhu S, Peng H, Chen Y, Cheng Z (2020). A small molecular compound CC1007 induces cross-lineage differentiation by inhibiting HDAC7 expression and HDAC7/MEF2C interaction in BCR-ABL1(-) pre-B-ALL. Cell Death Dis.

[45] Liu S, Li H, Chen L, Yang L, Li L, Tao Y, (2013). (-)-Epigallocatechin-3-gallate inhibition of Epstein-Barr virus spontaneous lytic infection involves ERK1/2 and PI3-K/Akt signaling in EBV-positive cells. Carcinogenesis.

