# Supplementary figures and images for: The JAK2 inhibitor TG101209 exhibits anti-tumor and chemotherapeutic sensitizing effects on Burkitt lymphoma cells by inhibiting the JAK2/STAT3/c-MYB signaling axis
Source: Cell Death Discov. 2021 Sep 29;7:268. doi: 10.1038/s41420-021-00655-1 (PMC8481535; doi:10.1038/s41420-021-00655-1)

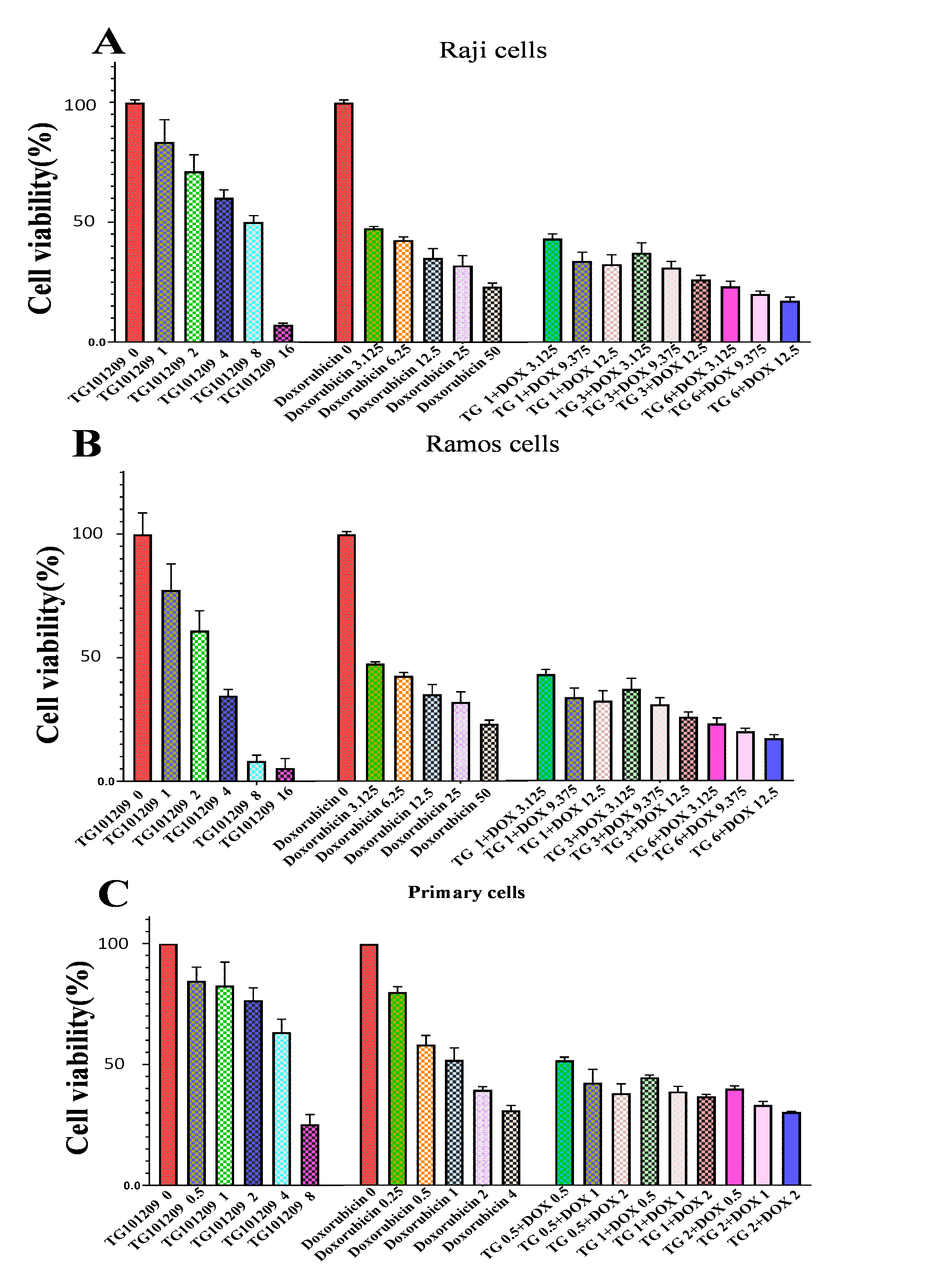

Supplement: Supplementary file 1 — Supplementary Figure [file 41420_2021_655_MOESM1_ESM.tif]
