# Supplementary material for: The JAK2 inhibitor TG101209 exhibits anti-tumor and chemotherapeutic sensitizing effects on Burkitt lymphoma cells by inhibiting the JAK2/STAT3/c-MYB signaling axis
Source: Cell Death Discov. 2021 Sep 29;7:268. doi: 10.1038/s41420-021-00655-1 (PMC8481535; doi:10.1038/s41420-021-00655-1)
Supplement: Supplementary file 2 — Supplementary Figure legend [file 41420_2021_655_MOESM2_ESM.docx]

**Supplementary Figure 1.** Inhibition of BL cells proliferation by TG101209 or DOX alone and in combination. Raji (A), Ramos (B) and primary BL cells (C) were treated with indicated concentration of TG101209 (0-16 μM) or doxorubicin (0-50μM) alone and in combination with different doses for 24 h, and growth inhibition was assessed using MTT.
