# Supplementary material for: The JAK2 inhibitor TG101209 exhibits anti-tumor and chemotherapeutic sensitizing effects on Burkitt lymphoma cells by inhibiting the JAK2/STAT3/c-MYB signaling axis
Source: Cell Death Discov. 2021 Sep 29;7:268. doi: 10.1038/s41420-021-00655-1 (PMC8481535; doi:10.1038/s41420-021-00655-1)
Supplement: Supplementary file 3 — Supplementary tables [file 41420_2021_655_MOESM3_ESM.docx]

Supplementary Table 1.

Combination index values of TG101209 and doxorubicin in Raji cells.

| Dose  TG101209(μM) | Dose [Doxorubicin](https://www.selleck.cn/products/Adriamycin.html)(μM) | Growth inhibiton(%) | Combinatio index(CI) |
| --- | --- | --- | --- |
| 1.0 | 3.125 | 56.75 | 0.76443 |
| 1.0 | 9.375 | 66.15 | 0.74917 |
| 1.0 | 12.5 | 67.48 | 0.82816 |
| 3.0 | 3.125 | 62.73 | 0.77596 |
| 3.0 | 9.375 | 69.00 | 0.8194 |
| 3.0 | 12.5 | 73.97 | 0.62658 |
| 6.0 | 3.125 | 76.83 | 0.625 |
| 6.0 | 9.375 | 79.94 | 0.59454 |
| 6.0 | 12.5 | 80.16 | 0.61654 |

Supplementary Table 2.

Combination index values of TG101209 and doxorubicin in Ramos cells.

| Dose  TG101209(μM) | Dose [Doxorubicin](https://www.selleck.cn/products/Adriamycin.html)(μM) | Growth inhibiton (%) | Combinatio index(CI) |
| --- | --- | --- | --- |
| 1.0 | 3.125 | 86.52 | 0.18044 |
| 1.0 | 9.375 | 79.44 | 0.77765 |
| 1.0 | 12.5 | 83.81 | 0.61816 |
| 3.0 | 3.125 | 92.12 | 0.24964 |
| 3.0 | 9.375 | 91.72 | 0.32117 |
| 3.0 | 12.5 | 90.75 | 0.39599 |
| 6.0 | 3.125 | 91.39 | 0.49341 |
| 6.0 | 9.375 | 95.86 | 0.36127 |
| 6.0 | 12.5 | 97.83 | 0.26802 |

Supplementary Table 3. Combination index values of TG101209 and

doxorubicin in Primary cells of Burkitt lymphoma.

| Dose  TG101209(μM) | Dose [Doxorubicin](https://www.selleck.cn/products/Adriamycin.html)(μM) | Growth inhibiton (%) | Combinatio index(CI) |
| --- | --- | --- | --- |
| 0.5 | 0.5 | 48.34 | 0.58465 |
| 0.5 | 1.0 | 57.52 | 0.64764 |
| 0.5 | 2.0 | 61.96 | 0.9516 |
| 1.0 | 0.5 | 55.34 | 0.49475 |
| 1.0 | 1.0 | 61.18 | 0.59881 |
| 1.0 | 2.0 | 63.20 | 0.94879 |
| 2.0 | 0.5 | 60.00 | 0.53306 |
| 2.0 | 1.0 | 66.78 | 0.5438 |
| 2.0 | 2.0 | 69.66 | 0.74066 |

Supplementary Table 4. Sequences of oligonucleotide primers used for RT-qPCR

| Gene | Sequence |
| --- | --- |
| Bcl-6 | fwd: 5΄- CCTGCAGATGGAGCATGTTG -3΄ |
|  | rev: 5΄- CATCAGCATCCGGCTGTTG -3΄ |
| c-Myb | fwd: 5΄- TGCTCCTAATGTCAACCGAGAAT -3΄ |
|  | rev: 5΄- ACAGGTGCACTGTCTCCATGAG -3΄ |
| PRDM1 | fwd: 5΄- TCCAGCACTGTGAGGTTTCA -3΄ |
|  | rev: 5΄- TCAAA CTCAGCCTCTGTCCA -3΄ |
| β-actin | fwd: 5΄-TTCCAGCCTTCCTTCCTGGG-3΄ |
|  | rev: 5΄-TTGCGCTCAGGAGGAGCAAT-3΄ |
