# Supplementary material for: The JAK2 inhibitor TG101209 exhibits anti-tumor and chemotherapeutic sensitizing effects on Burkitt lymphoma cells by inhibiting the JAK2/STAT3/c-MYB signaling axis
Source: Cell Death Discov. 2021 Sep 29;7:268. doi: 10.1038/s41420-021-00655-1 (PMC8481535; doi:10.1038/s41420-021-00655-1)
Supplement: Supplementary file 4 — Supplementary Information [file 41420_2021_655_MOESM4_ESM.docx]

**Supplementary Information**

**The JAK2 inhibitor TG101209 exhibits anti-tumor and chemotherapeutic sensitizing effects on on Burkitt lymphoma cells by inhibiting the JAK2/STAT3/c-MYB signaling axis**

**Materials and Methods**

**antibodies**

Antibodies used in the present study are as follows: The primary antibodies are as follows: p-JAK2 antibody (ab32101; Abcam, Cambridge, UK),β-actin (Ac-15; Sigma), the antibodies against Cleaved PARP antibody (#9541)，p-STAT3 (#9145)，Caspase 9 (#9502), Caspase 3 (#9665), bcl-6 (#5650s), PRDM1 (#9115s) , c-Myb (#12319s), Jak2 (#3230), Stat3 (#9139), Cyclin B1( #12231) , c-Myc (#9402) all from Cell Signaling Technology (Cell Signaling Technology Danvers, MA), antibodies against cytochrome c (sc-7159), HSP 60(sc-13115), goat anti-rabbit IgG-horseradish peroxidase (sc-2004) and goat anti-mouse IgG-horseradish peroxidase (sc-2005) all from Santa Cruz (Santa Cruz, CA).

**Immunohistochemical (IHC) staining**

IHC staining was performed as described previously. Briefly, tissue sections from xenograft tumor tissues were baked at 60 °C for 2 h, deparaffinized, and rehydrated. The slide was unmasked by submersion into boiling sodium citrate buffer (10 mM, pH 6.0) for 10 min, and then treated with 3% H2O2 for 10 min. The slide was blocked with 50% goat serum albumin in 1 × PBS in a humidified chamber for 1 h at room temperature. Primary antibody was incubated at 4 °C in a humidified chamber overnight. After hybridized with the second antibody for 45 min at room temperature, the DAB substrate was used for target protein visualization. Slides were viewed under a light microscope and analyzed.
